# Mutant structure of metabolic switch protein in complex with monomeric c-di-GMP reveals a potential mechanism of protein-mediated ligand dimerization

**DOI:** 10.1038/s41598-023-29110-0

**Published:** 2023-02-21

**Authors:** Badri Nath Dubey, Viktoriya Shyp, Geoffrey Fucile, Holger Sondermann, Urs Jenal, Tilman Schirmer

**Affiliations:** 1 Biozentrum, University of Basel, CH-4056 Basel, Switzerland; 2 CSSB Centre for Structural Systems Biology, Deutsches Elektronen-Synchrotron DESY, Notkestr. 85, 22607 Hamburg, Germany; 3 Department Research, University Center for Dental Medicine Basel UZB, University of Basel, Basel, Switzerland; 4 Department of Oral Health & Medicine, University Center for Dental Medicine Basel UZB, University of Basel, Basel, Switzerland; 5 sciCORE Center for Scientific Computing, University of Basel, CH-4056 Basel, Switzerland; 6 Christian-Albrechts-Universität zu Kiel, Kiel, Germany

**Keywords:** Biochemistry, Biophysics, Drug discovery, Microbiology, Molecular biology, Structural biology

## Abstract

Bacterial second messengers c-di-GMP and (p)ppGpp have broad functional repertoires ranging from growth and cell cycle control to the regulation of biofilm formation and virulence. The recent identification of SmbA, an effector protein from *Caulobacter crescentus* that is jointly targeted by both signaling molecules, has opened up studies on how these global bacterial networks interact. C-di-GMP and (p)ppGpp compete for the same SmbA binding site, with a dimer of c-di-GMP inducing a conformational change that involves loop 7 of the protein that leads to downstream signaling. Here, we report a crystal structure of a partial loop 7 deletion mutant, SmbA_∆loop_ in complex with c-di-GMP determined at 1.4 Å resolution. SmbA_∆loop_ binds monomeric c-di-GMP indicating that loop 7 is required for c-di-GMP dimerization. Thus the complex probably represents the first step of consecutive c-di-GMP binding to form an intercalated dimer as has been observed in wild-type SmbA. Considering the prevalence of intercalated c-di-GMP molecules observed bound to proteins, the proposed mechanism may be generally applicable to protein-mediated c-di-GMP dimerization. Notably, in the crystal, SmbA_∆loop_ forms a 2-fold symmetric dimer via isologous interactions with the two symmetric halves of c-di-GMP. Structural comparisons of SmbA_∆loop_ with wild-type SmbA in complex with dimeric c-di-GMP or ppGpp support the idea that loop 7 is critical for SmbA function by interacting with downstream partners. Our results also underscore the flexibility of c-di-GMP, to allow binding to the symmetric SmbA_∆loop_ dimer interface. It is envisaged that such isologous interactions of c-di-GMP could be observed in hitherto unrecognized targets.

## Introduction

In all domains of life, second messenger signaling is essential to modulate the intracellular response to external stimuli. In bacteria, purine nucleotide second messengers, such as guanosine tetra- and pentaphosphate, collectively referred to as (p)ppGpp, and bis-(3´-5´)-cyclic dimeric guanosine monophosphate, c-di-GMP, are involved in the global control of physiological responses to environmental change^1,2^. (p)ppGpp is the primary regulator of bacterial growth and development in response to stress and nutrient limitation also known as the stringent response^3–5^. It modulates cellular reprogramming via multiple target proteins including RNA polymerase, translational GTPases, and metabolic enzymes^6,7^, thereby controlling bacterial transcription, translation^8^, cell cycle progression^9,10^, stress resistance, and virulence^11,12^. In most bacteria, c-di-GMP controls the transition between motile and sessile lifestyles. Low c-di-GMP levels are associated with motility, while its accumulation promotes adhesion and biofilm formation^13–16^. However, an increasing number of studies indicate that c-di-GMP has an impact on diverse aspects of bacterial physiology including cell cycle progression, metabolism and stress resistance^2,17–22^.

The pleiotropic effects of (p)ppGpp and c-di-GMP are realized due to the diversity of their effectors, represented mainly by nucleotide-binding proteins and riboswitches^15,23,24^. In particular, the structural diversity of cyclic nucleotide, comprising various conformations from an extended monomeric form to a stacked dimer, explains the variety in c-di-GMP-binding motifs^25–27^. The canonical c-di-GMP binding sites are represented by RxxxR and [DN]xSxxG motifs in the PilZ domains, the RxxD motif in degenerate GGDEF I sites of DGCs and ExLxR in the EAL domains of PDEs. Moreover, several proteins with a non-canonical c-di-GMP binding motif have been recently characterized as high-affinity binding receptors, suggesting a widespread function of c-di-GMP in bacteria^25,28^.

**Figure 1:**
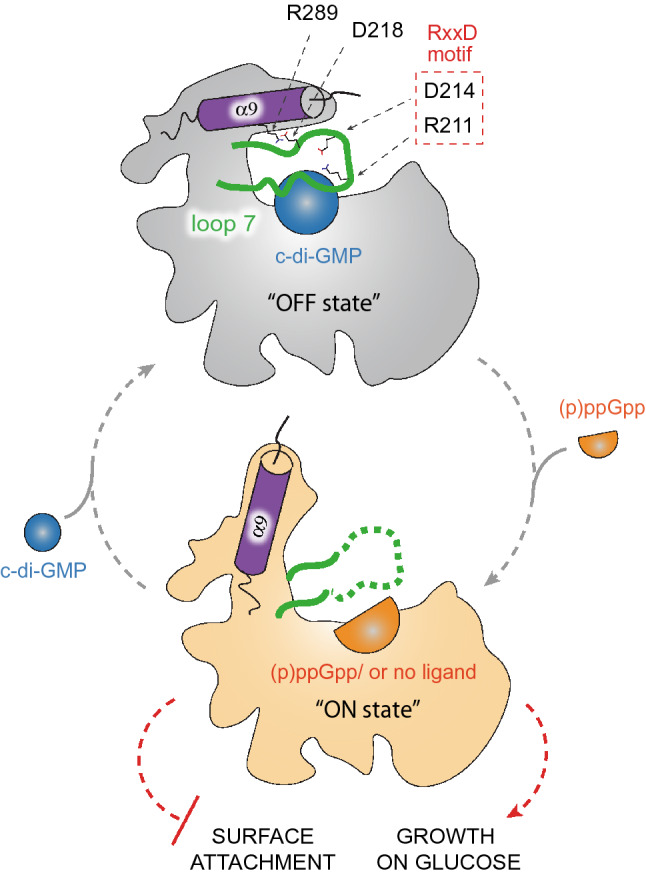
Second messenger mediated regulation of SmbA. Binding of a c-di-GMP dimer (blue sphere) inactivates SmbA (“OFF state”, grey), while its dissociation or displacement by a ppGpp monomer (an orange half-sphere) activates the protein (“ON state”, light orange). Loop 7 is shown in green, the C-terminal α9 helix is represented by a magenta cylinder. Amino acid residues essential for salt bridge formation between α9 helix and loop 7 are indicated. Key residues of the RxxD motif in loop 7 are shown in the red box. The physiological functions of activated SmbA are indicated with red dashed lines (Adopted from Shyp et al.^31^).

The development of biochemical methods to identify second messenger effectors greatly complemented our knowledge of novel c-di-GMP and/or (p)ppGpp binding proteins and their interaction networks^28–30^. Recently we have identified the first common target of c-di-GMP and ppGpp, SmbA protein from *C. crescentus*^31^. SmbA stimulates *Caulobacter* growth on glucose while preventing surface attachment in its active state repressed by binding of the c-di-GMP dimer (Fig. 1). The two ligands inversely regulate protein activity presumably by affecting its conformation. The major conformational changes promoting SmbA’s functional switch affect the C-terminal helix 9 and the flexible loop 7 containing c-di-GMP subsite residues R211 and D214 from the RxxD motif (Fig. 1). In the c-di-GMP-bound state, C-terminal helix 9 is stabilized by a salt bridge of D218 (from the loop7) and R289 (from helix 9), while in the ppGpp-bound state, loop 7 is disordered and helix 9 is in the open conformation (Fig. 1). Mutation of R211 to alanine leads to a prolonged adaptation phase and reduced growth in cells suggesting the involvement of loop 7 and potentially helix 9 in downstream signaling^31^.

To date, our structural knowledge about SmbA, however, is restricted to the wild-type protein in the presence of ligands. To understand how a flexible loop 7 influences the overall SmbA structure and its ligand binding, we present here the high-resolution structure of a loop 7 deletion mutant (fragment 198–215, hereafter SmbA_∆loop_). We observe that the mutant retains the TIM-barrel fold, however, accommodates only a monomer of c-di-GMP in a unique extended/open conformation. Importantly, in the SmbA_∆loop_ mutant, C-terminal helix 9 adopts an outward orientation similar to that found in the ppGpp-bound active state of the protein. Moreover, changes in c-di-GMP binding stoichiometry in SmbA_∆loop_ mutant, similar to loop 7 single mutant R211A, provide a potential mechanism and essential role of loop 7 in c-di-GMP dimerization and SmbA functional regulation.

## Results and discussion

### SmbA_∆loop_ forms a crystallographic dimer mediated by monomeric c-di-GMP

Ligand-induced conformational changes may be critical for SmbA physiological function, in particular for interaction with its yet-to-be-discovered downstream targets. Based on the fact that loop 7 is disordered in the apo-state but becomes ordered upon binding of a c-di-GMP dimer, and that mutation of the interacting arginine residue 211 from this loop renders SmbA inactive in signaling^31^, we hypothesize that loop 7 is a central component of the physiological switch.

To explore the structural changes promoted by c-di-GMP via loop 7 we tried to crystallize the apo form of SmbA protein as well as SmbA_R211A_ and a SmbA_∆loop_ mutant with a partial loop deletion (fragment 198–215 deleted) in complex with c-di-GMP. We only obtained suitable crystals for SmbA_∆loop_ (Supplementary Fig. S1a), which diffracted extremely well to 1.4 Å resolution and belong to space group P4_3_2_1_2 with one molecule in the asymmetric unit. The structure was determined by molecular replacement using the structure of wild-type SmbA (PDB: 6GS8^31^) after removing c-di-GMP from the model as a template, followed by iterative refinement. The data collection and refinement statistics are summarized in Table 1.

**Table 1:** Crystallographic data collection and refinement statistics.

Data collection	SmbA_Δloop_/c-di-GMP	SmbA_Δloop_
Synchrotron source	SLS, PXIII	SLS, PXI
Wavelength (Å)	1.00004	1.00004
Space group	P4_3_ 2_1_ 2	P 2_1_
a, b, c (Å)	56.0, 56.0, 205.1	61.4, 208.1, 64.2
α, β, γ (°)	90, 90, 90	90, 117.6, 90
Resolution (Å)	21.5–1.4 (1.45–1.4)*	56.9–1.8 (1.87–1.8)
Unique reflections	65,577 (6381)	128,620 (12,871)
Completeness	99.93 (99.87)	98.7 (97.9)
I/σ (I)	20.6 (2.6)	12.06 (2.9)
Redundancy	22.9 (22.9)	3.3 (3.3)
R_merge_ (%)	9.8 (164)	7.4 (55.5)
R_pim_ (%)	2.1 (352)	4.9 (36.1)
CC (1/2) %	99.9 (86.4)	99.6 (73.3)
**Refinement**		
R_work_/R_free_ (%)	14.8/17.7	16.8/20.3
**RMSD**		
Bond lengths (Å)	0.006	0.009
Bond angles (°)	0.9	1.05
Molecules/asymmetric unit	1	4
**No. of atoms**		
Protein	2081	8952
Ligand	99	0
Water	299	1265
Average B-factor (Å^2^)	20.4	24.0
Protein	18.5	22.7
Ligand	21.4	
Water	33.3	33.6
**Ramachandran statistics (%)**		
Favored regions	99.25	98.26
Allowed regions	0.75	1.74
Disallowed regions	0.0	0.0
**Deposition**		
PDB codes	7B0E	8BVB

(* = The values recorded in parentheses are those for the highest resolution shell).

**Figure 2:**
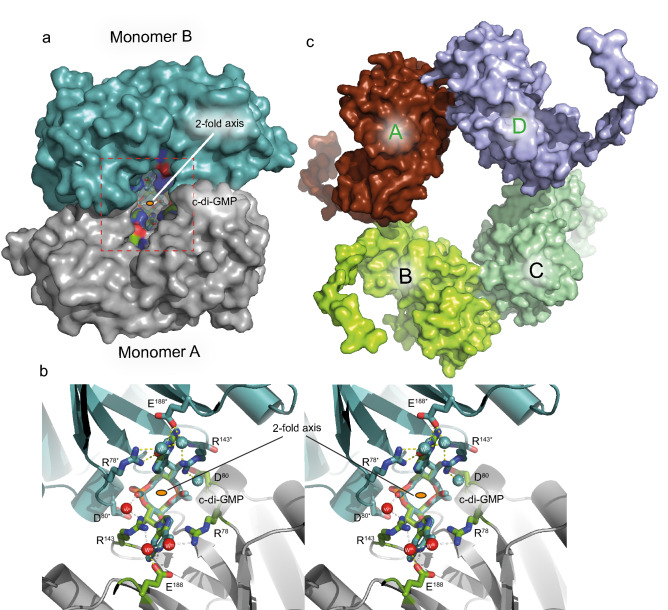
Crystal structures of SmbA_Δloop_ with c-di-GMP bound across the crystallographic dyad and of apo SmbA_Δloop_. (**a**) The two monomers are depicted as surface (negatively charged atoms in red, positively charged atoms in blue and carbon atoms in green) with monomer A (gray) in standard orientation and monomer B (symmetry mate) in cyan. c-di-GMP in the dimer interface is shown as ball-and-stick model. (**b**) Stereoview down the twofold axis (indicated as a small orange ellipsoid), showing c-di-GMP forming isologous interactions with the two SmbA_Δloop_ protomers. Relevant residues are shown as color-coded sticks (oxygen, red; nitrogen, blue; carbon, green or cyan and waters as red and cyan spheres) and labeled. Residues and waters of the symmetry mate monomer are marked with an asterisk. Hydrogen bonds between subunits and c-di-GMP are indicated as yellow dotted lines. (**c**) Crystal packing of apo SmbA_Δloop_ shown in surface representation. The four molecules are arranged in an asymmetric unit form two local dimers (A and D, B and C) with 2-fold symmetry.

The crystal structure shows that SmbA_∆loop_ forms a crystallographic dimer stabilized by a monomeric c-di-GMP molecule (Fig. 2a). The ligand is found in a fully extended conformation and makes isologous interactions with the two protomers of the protein dimer (Fig. 2b). The guanine bases of c-di-GMP interact extensively, via both polar and nonpolar contacts, with monomers A and B of the dimer. As in the wild-type complex, they form cation–π interactions with the guanidinium groups of R143 from both protomers (Fig. 2b). Detailed interactions will be discussed in detail further below.

In solution, the c-di-GMP to-protein stoichiometry using ITC was 1:1 (Supplementary Fig. S1b) and not 1:2 as would have been expected from the crystal structure, indication that SmbA_∆loop_ dimer formation occurs probably only at very high concentration as used for crystallization or during crystal formation.

**Figure 3:**
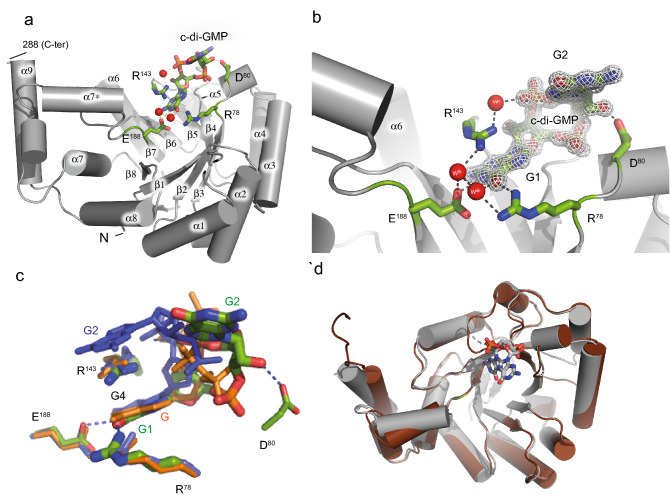
Detailled crystal structures of SmbA_Δloop_ in presence and absence of c-di-GMP and and structural comparison with wild-type SmbA ligands. (**a**) Crystal structure of the SmbA_Δloop_ with the backbone drawn in grey cartoon and monomeric c-di-GMP shown in a stick. Residues in the SmbA_Δloop_ important in interaction with the c-di-GMP molecule are drawn in stick representation. Carbon atoms are shown in green, nitrogen in blue and oxygen in red. (**b**) 2Fo-Fc omit maps contoured at 1.2 σ of c-di-GMP and full structural details of the interacting residues. H-bonds (length < 3.5 Å) are indicated by gray lines and water molecules in red spheres. (**c**) View of c-di-GMP (green) as bound to SmbA_Δloop_, and the proximal c-di-GMP molecule (blue) of dimeric c-di-GMP and ppGpp (orange) as bound to wild-type SmbA. The proximal guanyl of monomeric c-di-GMP (G1), guanyl of ppGpp (G) and G4 of dimeric c-di-GMP overlap closely. While the other guanyl (G2) of the monomeric ligand has moved out considerably, to form isologous interactions with the second SmbA_Δloop_ molecule (not shown). (**d**) structural superposition of SmbA_Δloop_/c-di-GMP (gray) with SmbA_Δloop_ (chocolate) yielding a RMSD of 0.49 Å.

In addition to the SmbA_∆loop_/c-di-GMP complex, we also determined a crystal structure of the protein in the absence of c-di-GMP. Overall, apo SmbA_∆loop_ shows virtually the same structure as in complex with c-di-GMP with an rmsd value of 0.49 Å for 225 Cα atoms (Fig. 3d). The Crystal contains four molecules in the asymmetric unit. Given the relatively small interface and loose packing in the crystal lattice, we consider the inter-molecular interactions to be crystallographic artifacts (Fig. 2c).

**Figure 4:**
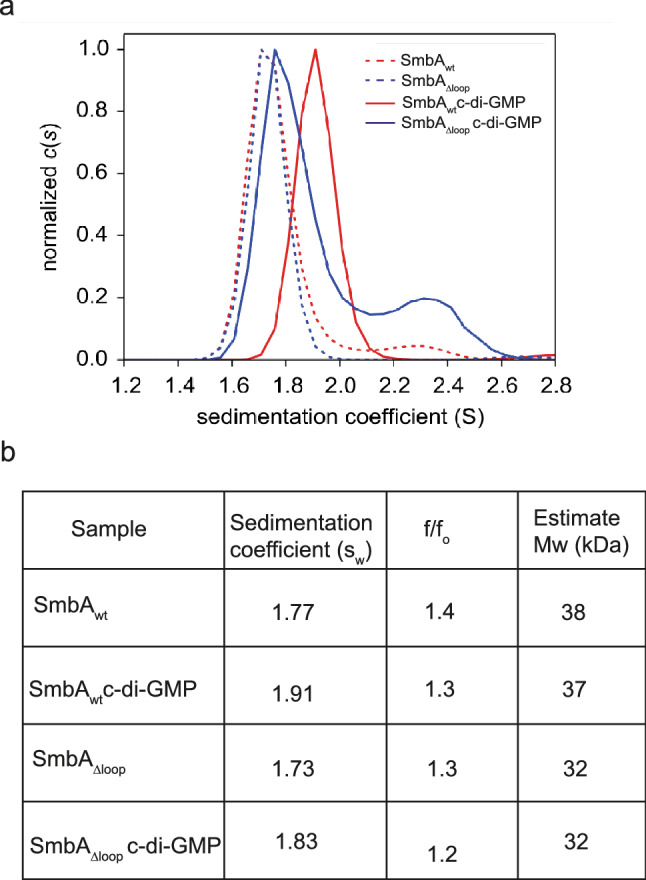
Analytical ultracentrifugation (AUC) analysis of SmbA_wt_ and SmbA_Δloop_. (**a**) SV-AUC absorbance c(s) distributions of SmbA_wt_, SmbA_wt_/(c-di-GMP)_2_, SmbA_Δloop_ and SmbA_Δloop_/c-di-GMP. (**b**) Mass estimation and s and f/f_O_ values of SmbA_wt_, SmbA_wt_/(c-di-GMP)_2_, SmbA_Δloop_ and SmbA_Δloop_/c-di-GMP.

As measured directly by sedimentation velocity analytical ultracentrifugation (AUC-SV), apo SmbA_∆loop_ is monomeric with a sedimentation coefficient of 1.73 s (Fig. 4). Addition of c-di-GMP does not change the sedimentation coefficient significantly. In addition, a small secondary peak is generated at 2.3 S, which may indicate some dimer formation. In contrast, SmbA_wt_ experiences a substantial shift in the sedimentation coefficient upon c-di-GMP addition, probably due to the larger mass of the dimeric ligand and the induced change in protein shape due to loop 7 ordering. As shown in Fig. 4b, a single species was observed in all cases with estimated masses were about 38 and 32 kDa for SmbA_wt_ and SmbA_∆loop,_ respectively. No significant difference in S and f/f_0_ upon addition of c-di-GMP was observed for both proteins (Fig. 4b). This result is consistent with our previous report that SmbA_wt_ does not change its oligomeric state upon c-di-GMP binding as derived from MALS data^31^. These results further support that in solution a single c-di-GMP molecule does not cause SmbA_∆loop_ to dimerization.

C-di-GMP-mediated dimer stabilization has been observed previously, involving dimeric, and tetrameric c-di-GMP in the case of VpsT^32^ and BldD^19^, respectively (for a review see ref. 27). Furthermore, c-di-GMP accommodation in the rigid dimer interface has been described for STING protein^33^. Notably, structure comparison shows that VpsT, STING, and SmbA involve symmetric stacking interactions (with W131, Tyr167, and R143, respectively) which cap two guanine bases of c-di-GMP from both sides at the dimer interface (Supplementary Fig. S2). We anticipate that protein dimerization involved c-di-GMP with isologous interactions may be operational in more, hitherto unrecognized target.

### Apo and c-di-GMP bound SmbA_∆loop_ structures and comparison with SmbA_wt_ structures

Overall, the SmbA_∆loop_ mutant retains the TIM-barrel fold with eight α-helices on the outside and eight parallel β-strands on the inside with an extra helix 9 (Fig. 3a). The occupancy of the c-di-GMP ligand was set to 50% to account for its binding across the crystallographic dyad (half of the c-di-GMP molecule belongs to the symmetry mate). The ligand fit to the electron density very well after considerable conformational adjustment of both guanine bases (Fig. 3b and supplementary Fig. S3). Thus, the mutant can accommodate only monomeric c-di-GMP, likely due to the absence of R211 and D214 of the RxxD motif of loop 7 essential for c-di-GMP dimer coordination (Fig. 3b). As discussed in the previous section, the monomeric c-di-GMP ligand forms isologous interaction with the two protomers of the dimer (Fig. 2). The interactions of each guanyl with the protein are the same as observed for the proximal guanyl moiety (G4) of dimeric c-di-GMP and G of ppGpp interacting with wild-type SmbA^31^ (Fig. 3c). R143 is found stacked upon the guanyl to form a cation–π interaction, R78 forms an H-bond with O6, and E188 forms on H-bond with N1 of the guanyl base (Fig. 3b and supplementary S3). Compared to the wild-type complex the phosphate has moved towards the protein and forms an H-bond with main-chain amide 80 (Fig. 3c). Three well-defined water molecules make hydrogen bonds with R78, E188, and R143 (Fig. 3c).

**Figure 5:**
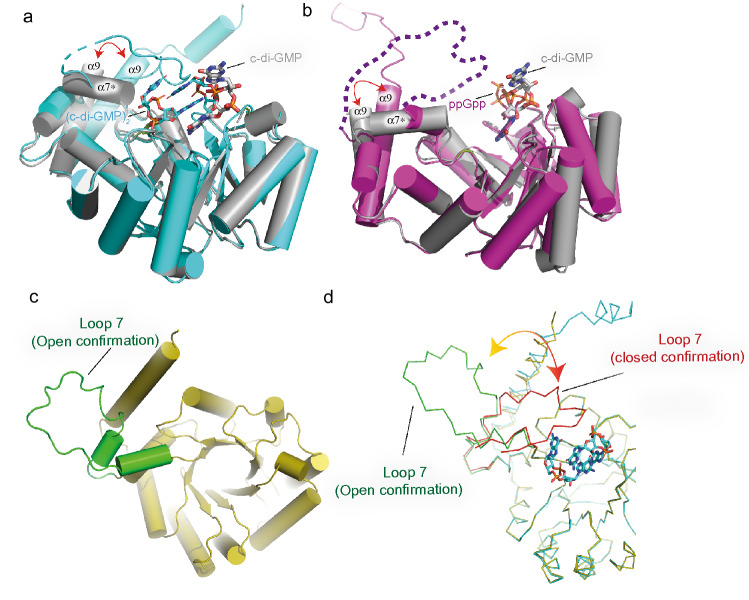
Structural comparison of SmbA_Δloop_/c-di-GMP with SmbA_wt_/(c-di-GMP)_2_, SmbA_wt_/ppGpp and Alphafold model of SmbA_wt_. (**a**) Superposition of SmbA_Δloop_/c-di-GMP (gray) with SmbA_wt_/(c-di-GMP)_2_ (cyan) with RMSD of 0.4. Relevant secondary structure elements are labeled. Dimeric c-di-GMP (cyan) and monomeric (thick) are shown as ball-and-stick models. (**b**) Superposition of SmbA_Δloop_/c-di-GMP (gray) with SmbA/ppGpp (Magenta) with RMSD of 0.5. Relevant secondary-structure elements are labeled. ppGpp (magenta) and monomeric (thick in gray) are shown as ball-and-stick models. The disordered part of loop 7 is marked by broken lines. (**c**) AlphaFold2 predicted model of SmbA_wt_ (yellow) with loop 7 is show in green color. (**d**) Superposition of SmbA_wt_/(c-di-GMP)_2_ (green) with AlphaFold2 model of SmbA_wt_ (yellow). Loop 7 from SmbA_wt_/(c-di-GMP)_2_ and Alphfold model of SmbA_wt_ apo are show in red and green repectively.

Structural superimposition of SmbA_∆loop_/c-di-GMP with SmbA_wt_/(c-di-GMP)_2 _(PDB code-6GS8) and SmbA_wt_/ppGpp (PDBcode-6GTM) shows RMS deviations of SmbA_∆loop_/c-di-GMP of 0.39 Å (for 214 Cα atoms) and 0.46 Å (for 230 Cα atoms) when compared to SmbA_wt_/(c-di-GMP)_2_ (Fig. 5b) and SmbA/ppGpp, respectively (Fig. 5b). These values indicate virtually idendical structures, but there are some notable local deviations. Particularly, in the SmbA_∆loop_/c-di-GMP complex, the C-terminal part of loop 7 forms a short helix α7* (Fig. 5a). In addition, significant changes are observed in the C-terminal helix 9, which, in the wild-type protein, is stabilized by loop 7 being in turn immobilized by dimeric c-di-GMP. Thereby, the G1 and G2 guanyl bases interact with the RxxD motif of loop 7^31^. In the SmbA_∆loop/_c-di-GMP complex, the monomeric ligand adopts an outward-open conformation similar to that found in SmbA_WT_/ppGpp complex (Fig. 5b). However, its guanyl is in the same position as the G of ppGpp and G4 of c-di-GMP all forming interactions with R78 and R143 (Figs. 3c and 5). At the same time, the phosphate moieties of monomeric c-di-GMP bound to SmbA_∆loop_ do not superimpose with those of bound dimeric c-di-GMP or ppGpp as bound to wild-type SmbA (Fig. 3c).

Because the apo structure of SmbA_wt_ is not known, we turned to a model of apo wild-type SmbA generated by AlphaFold2^34^ (AF2) as deposited in Uniprot (Q9A5E6) to predict the protein conformation and more specifically loop 7 in an unliganded state. The AF2 model of SmbA_wt_ agrees very well with our X-ray structure (Fig. 5d). Indeed, the core of the TIM-barrel fold shows very high confidence (pLDDT > 90) and represents the most stable region of the SmbA structure. Interestingly, loop 7 and helix 9 have low (70 > pLDDT > 50) and very low (pLDDT < 50) scores. It has been shown that AF2 correlated with the root mean square fluctuations (RMSF) calculated from MD (Molecular Dynamics) simulations experiments^35^. Thus the low AF2 scores of SmbA_wt_, suggest flexibility of loop 7 in the absence of the c-di-GMP (Supplementary Fig. S5a) which most likely is open in the unliganded state in contrast to closed in SmbA_wt_/(c-di-GMP)_2_ structure (Fig. 5c and d). This prediction is in line with the functional model of SmbA action^31^ which posits that, in response to c-di-GMP binding, the protein switches form an on- to off-state accompanied by structural changes in flexible loop 7 and helix 9, which ultimately controls the interaction with an unknown downstream partner possibly via heterodimerization (Supplementary Fig. S5b).

### Conformation of the monomeric c-di-GMP bound to SmbA_∆loop_

**Figure 6:**
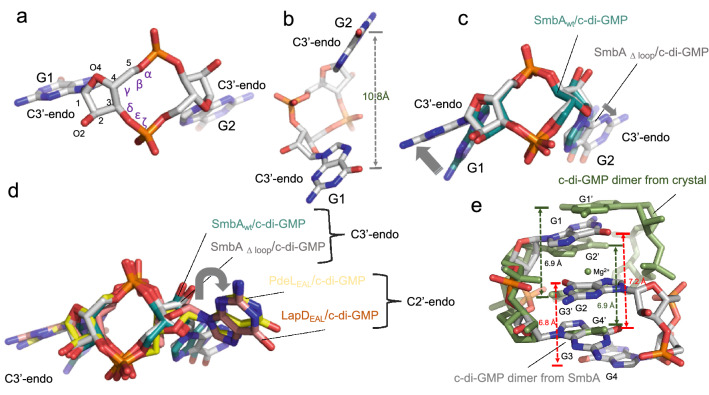
Observed c-di-GMP conformations in SmbA_loop_ and its comparison with SmbA_wt_, PdeL and LapD. (**a**) and (**b**) shows the partial open-twisted form of monomeric c-di-GMP in C3'-endo sugar pucker conformation observed in SmbA mutant. Guanine distances are shown in black dotted line. (**c**) Superimposition of c-di-GMP from SmbA_Δloop_, SmbA_wt_ and LapD_EAL_. GMP moiety from both structures shows the same conformation, the C3′-endo sugar pucker; however, there are considerable differences in the G1 and G2 base orientation (indicated by the gray arrow). (**d**) Superimposition of c-di-GMP from SmbA_Δloop_ with monomeric c-di-GMP as observed when bound to a phosphodiesterase PdeL and degenerated-phosphodiesterase LapD. Distinct sugar pucker of the base at the right (G2) appears responsible for the fully elongated form of c-di-GMP when bound to PdeL or LapD. In contrast, all bases at the left (G1) show the same sugar pucker, i.e. C3′-endo as also observed for SmbA_Δloop_ in this study. (**e**) Superimposition of crystal structure of c-di-GMP/Mg^2+^^36^ and dimeric c-di-GMP from SmbA_wt_. Guanine distances are shown in red and green dotted lines of c-di-GMP/Mg^2+^ and dimeric c-di-GMP from SmbA_wt_ respectevily.

As discussed above, with the deletion of the loop 7 containing the RxxD motif SmbA loses its ability to bind intercalated dimeric c-di-GMP molecule but still can hold one c-di-GMP. The monomeric ligand is two-fold symmetric, where the sugar pucker is C3′-endo, and both glycosidic torsion angles have a value of − 126° (Fig. 6a and b), which is significantly distinct to the *trans* conformation of G4 as part of dimeric c-di-GMP bound to wild-type SmbA. Superposition of c-di-GMP from the SmbA_∆loop_ and SmbA_wt_ complex structures shows that this difference is the reason for the elongated shape of monomeric c-di-GMP, while macrocycle including the sugar superimposes closely (Fig. 6c).

Next, we compared the conformation of monomeric c-di-GMP as bound to SmbA_**∆loop**_ to other effectors that bind the ligand in the monomeric form such as the phosphodiesterase domain PdeL_EAL_^37^ and the degenerate LapD_EAL_ domain^38^. A superposition of the three complexes is shown in Fig. 6d. While the macrocycles retain a similar, but not identical, conformation, as seen in the SmbA_∆loop,_ the ligands bound to PdeL_EAL_ and LapD_EAL,_ are in a more open conformation, apparently due to the C2′-endo puckering of one of the guanines (at the right side in Fig. 6d). These results show that c-di-GMP can adopt yet another unique conformation different from the stacked dimeric conformation in complex with SmbA_wt,_ or the extended form in the PdeL_EAL_ (PDB code-4LJ3) or degenerate LapD_EAL_ domain (PDB code-3PJT).

The monomeric c-di-GMP conformation observed in the SmbA_∆loop_ complex structure is different from that of dimeric c-di-GMP. From this comparison, one can see that one G1 is bound always the same way in the three complexes (Fig. 3c). Due to the conformational changes, the other GMP has moved out considerably, to form an isologous interaction with the second SmbA_∆loop_ molecule (interacting residues from monomer B is not shown) (Fig. 3c). This indicates that, depending on its binding partner, c-di-GMP is flexible enough to adopt various conformations via only minor changes in torsion-angle.

### The SmbA_Δloop_/c-di-GMP structure may represent the first step of consecutive c-di-GMP binding to form an intercalated dimer

At very high (> 1 mM) concentration, c-di-GMP can form dimers or even higher oligomers, such as tetramers or octamers. However, Gentner et al.^39^ clearly showed by NMR that c-di-GMP is monomeric at physiological concentrations. However it cannot be ruled out, but is unlikely, that other factors (metal ions, molecular crowding and aromatic compounds) may favor higher oligomers in the cellular environment. Intercalated c-di-GMP dimers have been observed in several protein complexes, such as when bound to the I-site of diguanylate cyclases, or in response regulators, PilZ receptors, and SmbA_wt_. Based on our data shown here, we propose that at physiological concentrations c-di-GMP dimerization occurs only on the protein by consecutive binding of c-di-GMP monomers to form the intercalated dimer (Fig. 6e).

This obviously implies the presence of a well formed, high-affinity protein binding site for the first c-di-GMP molecule. Here, we have captured upon loop deletion for the first time a potential binding pose of the first c-di-GMP binding event to SmbA. Indeed, all interactions required to bind this first monomer (involving R143, E188, R78) are present in SmbA_∆loop_ (Fig. 3c) and the affinity turned out to be in the low μM range (Fig. S1b). For the second binding event, in addition to a bound c-di-GMP molecule providing guanyl stacking sites, loop 7 providing the R_211_xxD_214_ motif would then be required (Supplementary Fig. S1e). In line with the structural considerations, the affinity of c-di-GMP to SmbA_∆loop_ is in the low micromolar range and is in fact comparable to the apparent K_d_ of c-di-GMP to the wild-type protein (Supplementary Fig. S1c and d).

In line with the structural considerations and the proposed binding mechanism, the K_d_ of c-di-GMP to SmbA_∆loop_ is low (1.8 μM) (Fig. S1b) and, in fact comparable, to the apparent K_d_ (0.3 μM) of the compound to the wild-type protein (Fig. S1c). For completeness, the affinity of ppGpp to the SmbA mutant was also measured (Fig. S1d) and was found to be virtually identical to the affinity of the compound to SmbA_w_^31^_t_ indicating that loop 7, as expected, does not contribute to ppGpp binding. In summary, the hypothesis of consecutive c-di-GMP binding to form an intercalated dimer on the protein is strongly supported by the results on the SmbA loop deletion mutant.

### Phylogenetic analysis and exploring SmbA homologs

**Figure 7:**
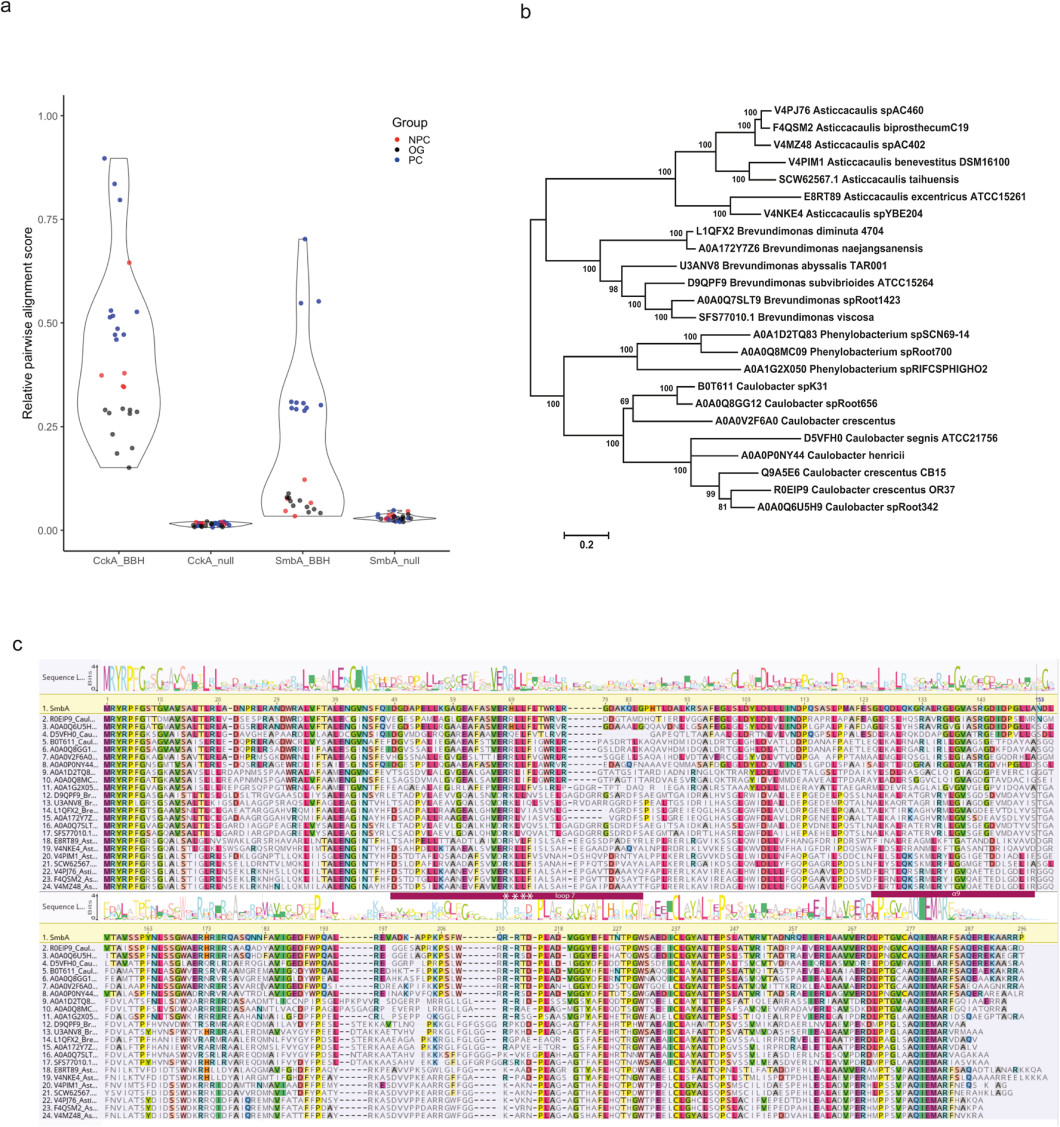
Sequence alignment and distance of SmbA homologs. (**a**) Pairwise Needleman-Wunsch global alignment scores of SmbA and CckA reciprocal best BLAST hits (BBH) for species sampled from prosthecate Caulobacterales (PC), non-prosthecate Caulobacterales (NPC), and other bacterial groups (OG). Alignment scores are reported relative to self-alignment of SmbA (Q9A5E6) and CckA (H7C7G9) from Caulobacter crescentus. For the null models, CckA BBH was scored against SmbA and vice versa. The latter BBH was identified using BLASTp against the NCBI-NR database using the BLOSUM45 scoring matrix. (**b**) A phylogenetic tree of 24 SmbA orthologs inferred using the Maximum Likelihood method based on the JTT model as implemented in MEGA7. Branch lengths indicate the number of substitutions per site. The tree with the highest log likelihood (− 9134.38) is shown, with bootstrap support from 100 replicates indicated at branches. (**c**) Sequence alignment and logo of SmbA orthologs. The sequence logo was generated using the WebLogo server from the global alignment of SmbA orthologs used to build the distance tree.

To understand the evolutionary significance of the flexible loop of SmbA switch protein, here we have further extended our primary sequence analysis of SmbA and its homologs described in Shyp et al.^31^. We identified SmbA orthologs based on reciprocal best BLAST hits across species, concordance of the protein sequence distance tree with a species phylogeny based on 16S rRNA markers^40^ and syntenic conservation^41^ (Fig. 7a and b). Interestingly, the c-di-GMP-binding RxxD motif is only strictly conserved within the *Caulobacter* genus, with either Asn or Glu substitution among the *Caulobacterales* (Fig. 7c). There is considerable variability around this loop region, including several insertions and deletion events. This may suggest alternative binding modes and/or substrates within the *Caulobacterales* order. Similarly, the sites interacting with ppGpp (R78, N111, Q114, R143, E188) are not strictly conserved within the *Caulobacterales* order. The C-terminal helix 9 is highly conserved among SmbA orthologs (Fig. 7c). This is consistent with the proposal that it adopts a different conformation in the c-di-GMP-bound state than apo and ppGpp, thus necessary for the ligand-mediated SmbA switch. A similar mechanism may apply to other SmbA orthologs via interplay of unknown ligands. The strictly conserved N-terminal motif (MRYRP[FL]G) is also found in otherwise unrelated proteins from the *Acetomycetalesorder* (Frankia, Streptomyces).

The *Caulobacterales* order contains prosthecate and non-prosthecate species^40,42^. SmbA (Q9A5E6) appears to be unique to the prosthecate *Caulobacterales*. Reciprocal best BLAST hits for SmbA from non-prosthecate *Caulobacterales* and other bacterial species are very distantly related Aldo–keto reductases which cannot be meaningfully aligned with SmbA. The central function of SmbA is a simple molecular switch that responds to the cellular concentrations of ppGpp and c-di-GMP to regulate *Caulobacter* growth^31^. We surmise that the presence of a SmbA ortholog is a marker for prosthecate-type *Caulobacterales* species which have not been morphologically characterized. This is further supported by the genes flanking SmbA, including a putative iron-sulfur glutaredoxin (Q9A5E5) and a BolA/YrbA family transcription factor (Q9A5E7) which in *E. coli* positively regulates the transition from the planktonic to attachment stage of biofilm formation^43^.

## Methods

### Plasmid construction and purification of the recombinant proteins

To construct pET21b-smbA_∆loop_-His6 (deletion of fragment 198–215), the pET21b-smbA-His6 plasmid was amplified with the following primers: 6265_D Loop7_forward CCCCAGGCCCTGCGAGAACTGGCCGATGTGGGCGGCTA and 6266_DLoop7_reverse TAGCCGCCCACATCGGCCAGTTCTCGCAGGGCCTGGGG. The template was digested with DpnI and mutant DNA was transformed into competent cells for nick repair. The final construct has been sequenced to confirm the fragment deletion. Protein was overproduced and purified as described previously^31^. *E. coli* Rosetta 2(DE3) cells were used to overproduce recombinant protein from the pET21b expression plasmid. Cells were grown in LB-Miller supplemented with 100 μg/ml ampicillin to an OD_600_ of 0.4–0.6, expression was induced with 1 mM IPTG overnight at 22 °C. Cells were harvested by centrifugation (5000 g, 20 min, 4 °C), washed with PBS and flash-frozen in liquid N_2_, and stored at − 80 °C until purification.

For purification, cells were resuspended in *lysis buffer* (30 mM Tris/HCl pH 7.5, 5 mM MgCl_2_, 100 mM NaCl, 1 mM DTT and 10 mM imidazole containing 0.2 mg/ml lysozyme, DNaseI (AppliChem) and Complete Protease inhibitor (Roche) and disrupted using a French press. The suspension was clarified by centrifugation at 30,000 × g (Sorval SLA 1500) at 4 °C for 30 min and loaded onto a 1 ml HisTrap HP column (GE Healthcare) on an ÄKTA purifier 10 system (GE Healthcare). Column was washed with 5 column volumes with *wash buffer* (30 mM Tris/HCl pH 7.5, 5 mM MgCl_2_, 100 mM NaCl, 1 mM DTT and 10 mM imidazole), and the bound protein was eluted with linear gradient of *elution buffer* (30 mM Tris/HCl pH 7.5, 3 mM MgCl_2_, 100 mM NaCl, 1 mM DTT and 300 mM imidazole). Elution fractions enriched in SmbA (as judged by SDS-PAGE) were pooled and concentrated to around 10 mg/ml using Amicon Ultra centrifugal concentrator with a nominal molecular weight cut-off of 30 kDa (Millipore AG). The concentrated protein was centrifuged at 16,000 × g at 4 °C for 15 min and loaded onto a Superdex 75 gel filtration column (Amersham Biosciences) equilibrated with 30 mM Tris/HCl pH 7.5, 5 mM MgCl_2_, 100 mM NaCl, 1 mM DTT. Fractions containing essentially pure SmbA (as judged by SDS-PAGE) were pooled and concentrated to a desired concertation for further experiments.

### Crystallization

A Phoenix robot (Art Robbins Instruments) was used for a wide range of crystallization screening. Crystallization was carried out using the sitting drop vapour diffusion method at 20 °C by mixing the protein with the reservoir solution in a 1:1 ratio. The protein concentration was 5.0, 2.25 and 1.75 mg/ml upon adding c-di-GMP in 3.0 fold molar excess. Triangle diamond-shaped 3D crystals appeared in Pact premier D11 (Molecular dimension) after one week in 0.2 M Calcium chloride dihydrate 0.1 M Tris pH 8.0 and 20% w/v PEG 6000. Crystals were flash-frozen into two different cryoprotectants. The best diffraction was obtained from crystals cryo-protected with 25% ethylene glycol.

For the apo protein crystals, three different protein concentrations (20, 15 and 5 mg/ml) were used at room temperature. Crystals appeared within a week and continued growoing for a few additional days in a condition containing 200 mM NaCl and 10% v/w PEG 6000. The crystals were flash-frozen in liquid N2 for data collection at 100 K.

### X-Ray diffraction data collection, phasing, and refinement

All single-crystal X-ray diffraction data sets were collected at PXI and PXIII beamline of Swiss Light source, Villigen, Switzerland.) Datasets were collected for the crystal of the SmbA_∆loop_ apo and in presence of c-di-GMP. Diffraction data sets were processed either with MOSFLM^44^ or XDS^45^ and the resulting intensities were scaled using SCALA from CCP4/CCP4i2 suite^46^For solving the SmbA_∆loop_ apo and complex structure, SmbA_wt_ (PDB code, 6GS8) structure was used as search model without c-di-GMP. Both structures were solved by molecular replacement using PHENIX PHASER^47^. Further refinement of structures was carried out using REFMAC5 and Phenix refinement^48^. Model building was performed using COOT^49^ and model validation was carried out with molprobity^50^. Crystallographic data processing and refinement statistics are provided in Table 1.

### Isothermal titration calorimetry (ITC)

Experiments were carried out at 25 °C or 10 °C, a syringe stirring speed of 300 rpm, a pre-injection delay of 200 secs, and a recording interval of 250 secs in a Microcal VP-ITC in ITC buffer (30 mM Tris–HCl pH 7.5, 150 mM NaCl, 5 mM MgCl_2_). All solutions were degassed below the temperature used in the experiments before loading into the calorimeter cell. Baseline correction and integration of the raw differential power data, and fitting of the resulting binding isotherms to obtain dissociation constants were performed using the Microcal ORIGIN software.

### Analytical ultracentrifugation (AUC)

Sedimentation velocity (SV) centrifugation was performed on a ProteomLab™ XL-A analytical ultracentrifuge (Beckman-Coulter, Brea, CA, USA) using an AN60 Ti rotor with standard aluminum 2-channel centerpieces with quartz windows. The samples were spun at speeds ranging from 35,000 to 50,000 rpm depending on the protein size at 4 °C. The SmbA_wt_ (38.9 μM) and SmbA_∆loop_ (39.0 μM) in SEC buffer was subjected to ultracentrifugation in the absence and in presence of a fivefold molar excess of c-di-GMP. Radial scans were recorded with 30 µm radial resolution at ~ 3 min intervals. The software packages SEDFIT v 14.14 was used for data evaluation. After transformation of the recorded sedimentation velocity data taken in the intensity mode to interference data in the respective data evaluation software, time- as well as radially-invariant noise were calculated and subtracted. In SEDFIT (http://www.analyticalultracentrifugation.com), continuous sedimentation coefficient distributions c(s) were determined with 0.05 S resolution and an F-ratio = 0.95. Suitable s-value ranges between 0 and 30 S and for GA f/f_0_ between 1 and 4 were chosen. Buffer density (1.0136 g/ml) and viscosity (1.591 cP) were calculated with SEDNTERP v 20111201 beta (http://bitcwiki.sr.unh.edu/index.php). The partial specific volumes of the studied proteins were calculated according to the method of Cohn and Edsall as implemented in SEDNTERP. From the peak in the c(s) distribution, the frictional ratio f/f0 and the meolecular weight were obtained by SEDFIT based on the Stokes–Einstein and Svedberg equations^51^. Data were plotted using program ProFit (Quansoft, Zurich, Switzerland).

### AlphaFold modeling

The SmbAwt AphaFold model was retrieved from Uniprot (https://www.uniprot.org) with accession code Q9A5E6. The X-ray structures were visualized using Pymol (https://pymol.org/2/) and compared to the AlphaFold model.

### Bioinformatics

BLAST analyses were conducted using the NCBI-NR dataset. Multiple sequence alignments were generated using MAFFT in G-INS-i mode^52^ followed by manual refinement. The phylogenetic tree of 24 SmbA orthologs was inferred using the Maximum Likelihood method based on the JTT model^53^ as implemented in MEGA7^54^. Branch lengths indicate the number of substitutions per site. The tree with the highest log likelihood (− 9134.38) is shown, with bootstrap support from 100 replicates indicated at branches. Initial tree(s) for the heuristic search were obtained automatically by applying Neighbor-Join and BioNJ algorithms to a matrix of pairwise distances estimated using a JTT model, and then selecting the topology with a superior log-likelihood value. A discrete Gamma distribution was used to model evolutionary rate differences among sites (5 categories, + G = 2.2328)). The rate variation model allowed for some sites to be evolutionarily invariable ([+ I], 7.16% sites). The tree is drawn to scale, with branch lengths measured in the number of substitutions per site. There were a total of 323 positions in the final dataset.

## Supplementary Information

Supplementary Information.

## Data Availability

The final SmbA_∆loop_ coordinates and structure factor amplitudes have been deposited in the Protein Data Bank (PDB) and are available under accession number 7B0E (SmbA_∆loop_/c-di-GMP) and 8BVB (SmbA_∆loop_).

## References

[1] Hauryliuk V, Atkinson GC, Murakami KS, Tenson T, Gerdes K (2015). Recent functional insights into the role of (p)ppGpp in bacterial physiology. Nat. Rev. Microbiol..

[2] Jenal U, Reinders A, Lori C (2017). Cyclic di-GMP: Second messenger extraordinaire. Nat. Rev. Microbiol..

[3] Cashel M, Gallant J (1969). Two compounds implicated in the function of the RC gene of *Escherichia coli*. Nature.

[4] Dalebroux ZD, Swanson MS (2012). ppGpp: Magic beyond RNA polymerase. Nat. Rev. Microbiol..

[5] Kalia D (2012). Nucleotide, c-di-GMP, c-di-AMP, cGMP, cAMP, (p)ppGpp signaling in bacteria and implications in pathogenesis. Chem. Soc. Rev..

[6] Potrykus K, Murphy H, Philippe N, Cashel M (2011). ppGpp is the major source of growth rate control in *E. coli*. Environ. Microbiol..

[7] Stott KV (2015). (p)ppGpp modulates cell size and the initiation of DNA replication in *Caulobacter crescentus* in response to a block in lipid biosynthesis. Microbiology.

[8] Wood, A., Irving, S., Bennison, D. & Corrigan, R. The (p)ppGpp-binding GTPase Era promotes rRNA processing and cold adaptation in *Staphylococcus aureus*. *Access Microbiol.* **2** (2020). 10.1371/journal.pgen.1008346 PMC6738653 31465450

[9] Gonzalez D, Collier J (2014). Effects of (p)ppGpp on the progression of the cell cycle of *Caulobacter crescentus*. J. Bacteriol..

[10] Lesley JA, Shapiro L (2008). SpoT regulates dnaa stability and initiation of DNA replication in carbon-starved *Caulobacter crescentus*. J. Bacteriol..

[11] Martins D (2018). Superoxide dismutase activity confers (p)ppGpp-mediated antibiotic tolerance to stationary-phase *Pseudomonas aeruginosa*. Proc. Natl. Acad. Sci. U. S. A..

[12] Nguyen D (2011). Active starvation responses mediate antibiotic tolerance in biofilms and nutrient-limited bacteria. Science.

[13] Boehm A (2010). Second messenger-mediated adjustment of bacterial swimming velocity. Cell.

[14] Matsuyama BY (2016). Mechanistic insights into c-di-GMP–dependent control of the biofilm regulator FleQ from *Pseudomonas aeruginosa*. Proc. Natl. Acad .Sci..

[15] Baraquet C, Harwood CS (2013). Cyclic diguanosine monophosphate represses bacterial flagella synthesis by interacting with the Walker A motif of the enhancer-binding protein FleQ. Proc. Natl. Acad. Sci..

[16] Fazli M (2014). Regulation of biofilm formation in Pseudomonas and Burkholderia species. Environ. Microbiol..

[17] Morgan JLW, McNamara JT, Zimmer J (2014). Mechanism of activation of bacterial cellulose synthase by cyclic-di-GMP. Nat. Struct. Mol. Biol..

[18] Römling U, Gomelsky M, Galperin MY (2005). C-di-GMP: The dawning of a novel bacterial signalling system. Mol. Microbiol..

[19] Tschowri N (2014). Tetrameric c-di-GMP mediates effective transcription factor dimerization to control streptomyces development. Cell.

[20] Lori C (2015). Cyclic di-GMP acts as a cell cycle oscillator to drive chromosome replication. Nature.

[21] Dubey BN (2016). Cyclic di-GMP mediates a histidine kinase/phosphatase switch by noncovalent domain cross-linking. Sci. Adv..

[22] Dubey BN (2020). Hybrid histidine kinase activation by cyclic di-GMP–mediated domain liberation. Proc. Natl. Acad. Sci..

[23] Li X, Chen F, Xiao J, He J (2017). Structure and function of c-di-GMP riboswitches. Sheng Wu Gong Cheng Xue Bao Chin. J. Biotechnol..

[24] Leduc JL, Roberts GP (2009). Cyclic di-GMP allosterically inhibits the CRP-like protein (Clp) of *Xanthomonas axonopodis* pv. citri. J. Bacteriol..

[25] Krasteva PV, Sondermann H (2017). Versatile modes of cellular regulation via cyclic dinucleotides. Nat. Chem. Biol..

[26] Hengge R (2009). Principles of c-di-GMP signalling in bacteria. Nat. Rev. Microbiol..

[27] Chou S-H, Galperin MY (2016). Diversity of cyclic Di-GMP-binding proteins and mechanisms. J. Bacteriol..

[28] Wang Y-C (2016). Nucleotide binding by the widespread high-affinity cyclic di-GMP receptor MshEN domain. Nat. Commun..

[29] Nesper J, Reinders A, Glatter T, Schmidt A, Jenal U (2012). A novel capture compound for the identification and analysis of cyclic di-GMP binding proteins. J. Proteom..

[30] Haas TM (2022). Photoaffinity capture compounds to profile the magic spot nucleotide interactomes**. Angew. Chem. Int. Ed..

[31] Shyp V (2021). Reciprocal growth control by competitive binding of nucleotide second messengers to a metabolic switch in *Caulobacter crescentus*. Nat. Microbiol..

[32] Krasteva PV (2010). Vibrio cholerae VpsT regulates matrix production and motility by directly sensing cyclic di-GMP. Science.

[33] Shang G (2012). Crystal structures of STING protein reveal basis for recognition of cyclic di-GMP. Nat. Struct. Mol. Biol..

[34] Jumper J (2021). Highly accurate protein structure prediction with AlphaFold. Nature.

[35] Guo H-B (2022). AlphaFold2 models indicate that protein sequence determines both structure and dynamics. Sci. Rep..

[36] Egli M (1990). Atomic-resolution structure of the cellulose synthase regulator cyclic diguanylic acid. Proc. Natl. Acad. Sci..

[37] Sundriyal A (2014). Inherent regulation of EAL domain-catalyzed hydrolysis of second messenger cyclic di-GMP*. J. Biol. Chem..

[38] Navarro MVAS (2011). Structural basis for c-di-GMP-mediated inside-out signaling controlling periplasmic proteolysis. Plos Biol..

[39] Gentner M, Allan MG, Zaehringer F, Schirmer T, Grzesiek S (2012). Oligomer formation of the bacterial second messenger c-di-GMP: Reaction rates and equilibrium constants indicate a monomeric state at physiological concentrations. J. Am. Chem. Soc..

[40] Abraham W-R (2008). Phylogeny by a polyphasic approach of the order Caulobacterales, proposal of *Caulobacter mirabilis* sp. nov., *Phenylobacterium haematophilum* sp. nov., and *Phenylobacterium conjunctum* sp. nov., and emendation of the genus *Phenylobacterium*. Int. J. Syst. Evol. Microbiol..

[41] Altenhoff AM (2015). The OMA orthology database in 2015: Function predictions, better plant support, synteny view and other improvements. Nucleic Acids Res..

[42] Abraham W-R, Rohde M, Bennasar A (2014). The Prokaryotes: Alphaproteobacteria and Betaproteobacteria.

[43] Dressaire C, Moreira RN, Barahona S, de Matos APA, Arraiano CM (2015). BolA is a transcriptional switch that turns off motility and turns on biofilm development. MBio.

[44] Leslie AGW (2006). The integration of macromolecular diffraction data. Acta Crystallogr. Sect. D Biol. Crystallogr..

[45] Kabsch W (2010). XDS. Acta Crystallogr. Sect. D Biol. Crystallogr..

[46] Potterton L (2018). CCP4i2: The new graphical user interface to the CCP4 program suite. Acta Crystallogr. Sect. D Struct. Biol..

[47] McCoy AJ (2007). Phaser crystallographic software. J. Appl. Crystallogr..

[48] Terwilliger TC (2008). Iterative model building, structure refinement and density modification with the PHENIX AutoBuild wizard. Acta Crystallogr. Sect. D Biol. Crystallogr..

[49] Emsley P, Cowtan K (2004). Coot: Model-building tools for molecular graphics. Acta Crystallogr. Sect. D Biol. Crystallogr..

[50] Williams CJ (2018). MolProbity: More and better reference data for improved all-atom structure validation. Protein Sci..

[51] Brown PH, Schuck P (2006). Macromolecular size-and-shape distributions by sedimentation velocity analytical ultracentrifugation. Biophys. J..

[52] Katoh K, Kuma K, Toh H, Miyata T (2005). MAFFT version 5: Improvement in accuracy of multiple sequence alignment. Nucleic Acids Res..

[53] Jones DT, Taylor WR, Thornton JM (1992). The rapid generation of mutation data matrices from protein sequences. Bioinformatics.

[54] Kumar S, Stecher G, Tamura K (2016). MEGA7: Molecular evolutionary genetics analysis version 7.0 for bigger datasets. Mol. Biol. Evol..

