# Supplementary material for: Mutant structure of metabolic switch protein in complex with monomeric c-di-GMP reveals a potential mechanism of protein-mediated ligand dimerization
Source: Sci Rep. 2023 Feb 21;13:2727. doi: 10.1038/s41598-023-29110-0 (PMC9944927; doi:10.1038/s41598-023-29110-0)
Supplement: Supplementary file 1 — Supplementary Information. [file 41598_2023_29110_MOESM1_ESM.pdf]

Supplementary information for:

Mutant structure of metabolic switch protein in complex with monomeric c-di-GMP reveals a potential mechanism of protein mediated ligand dimerization

Badri Nath Dubey, Viktoriya Shyp, Geoffrey Fucile, Holger Sondermann, Urs Jenal and Tilman Schirmer

Contents:

Figure S1 **$SmbA_{\Delta loop}$ crystals and binding parameters of $SmbA_{wt}$ and $SmbA_{\Delta loop}$ with c-di-GMP and ppGpp** 2-3

Figure S2 **c-di-GMP bound at dimer interface found in STING and VpsT** 4

Figure S3 **An expanded stereo view of the $SmbA_{\Delta loop}$ residues interacting with c-di-GMP** 5

Figure S4 **Absorbance data from the sedimentation velocity experiments of SmbA varients** 6

Figure S5 **AlphaFold model of $SmbA_{wt}$ and proposed model of downstream signaling of SmbA** 7

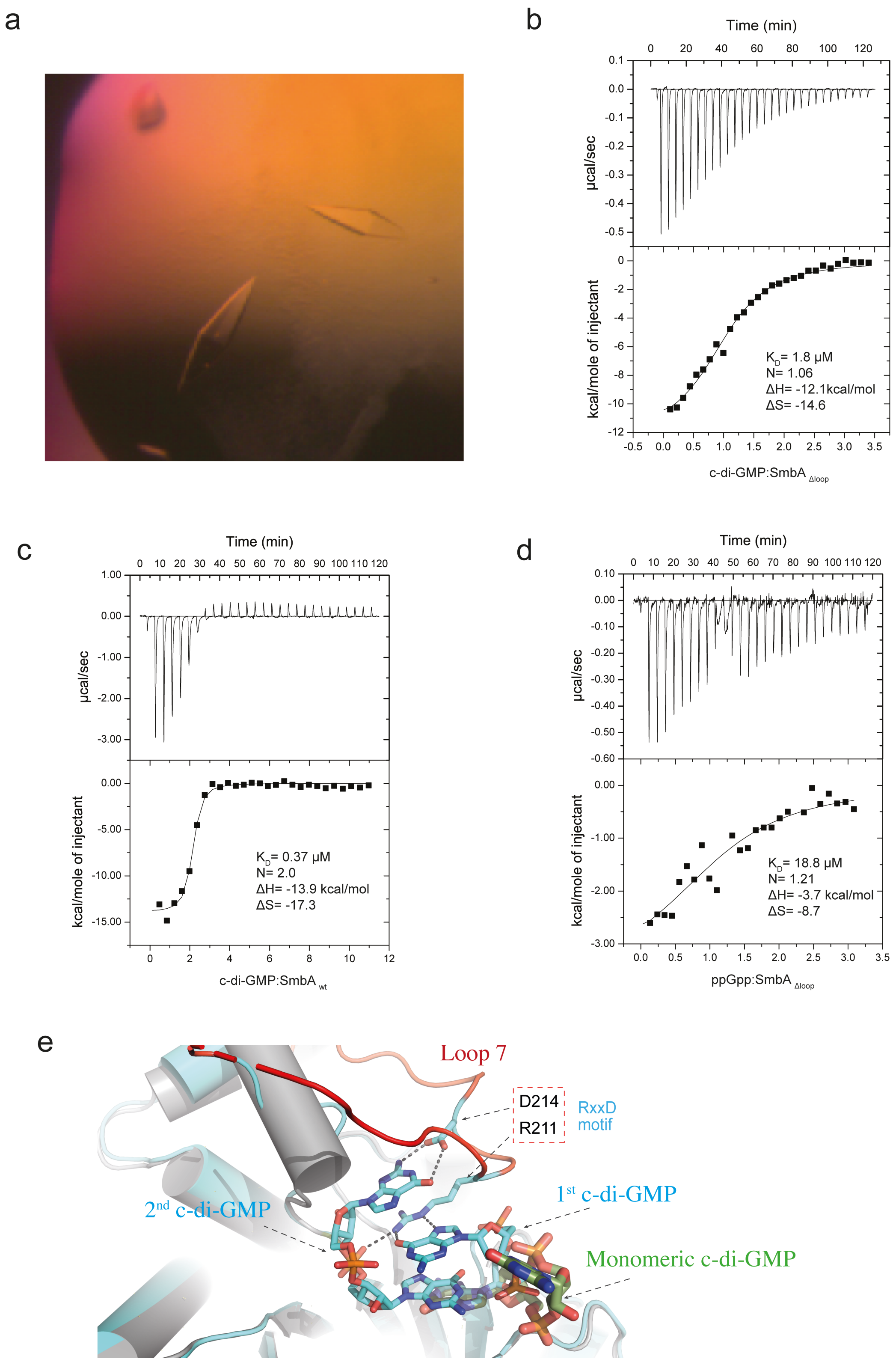

Figure S1. $SmbA_{\Delta loop}$ crystals and binding parameters of $SmbA_{wt}$ and $SmbA_{\Delta loop}$ with c-di-GMP and ppGpp. **(a)** $SmbA_{\Delta loop}$ crystals in complex with c-di-GMP.**(b)** ITC of $SmbA_{\Delta loop}$ (10 μM) binding to the c-di-GMP molecule (150 μM). The binding stoichiometry, ΔH, ΔS, and K_D are marked. **(c)** and **(d)** Isotherms representing binding of $SmbA_{wt}$ (15 μM) with c-di-GMP (800 μM) and $SmbA_{\Delta loop}$ (30 μM) ppGpp (500 μM), measured by ITC. The K_d, ΔH, ΔS and complex stoichiometry (*N*) values are indicated. **(e)** Superimposition of monomeric c-di-GMP (green) as bound to $SmbA_{\Delta loop}$, and dimeric c-di-GMP molecule (cyan) as bound to wild-type SmbA. I^{st} c-di-GMP of dimeric c-di-GMP overlap

closely with monomeric c-di-GMP. $SmbA_{\Delta loop}$ failed to secure the 2nd c-di-GMP due to a lack of RxxD residues of loop7.

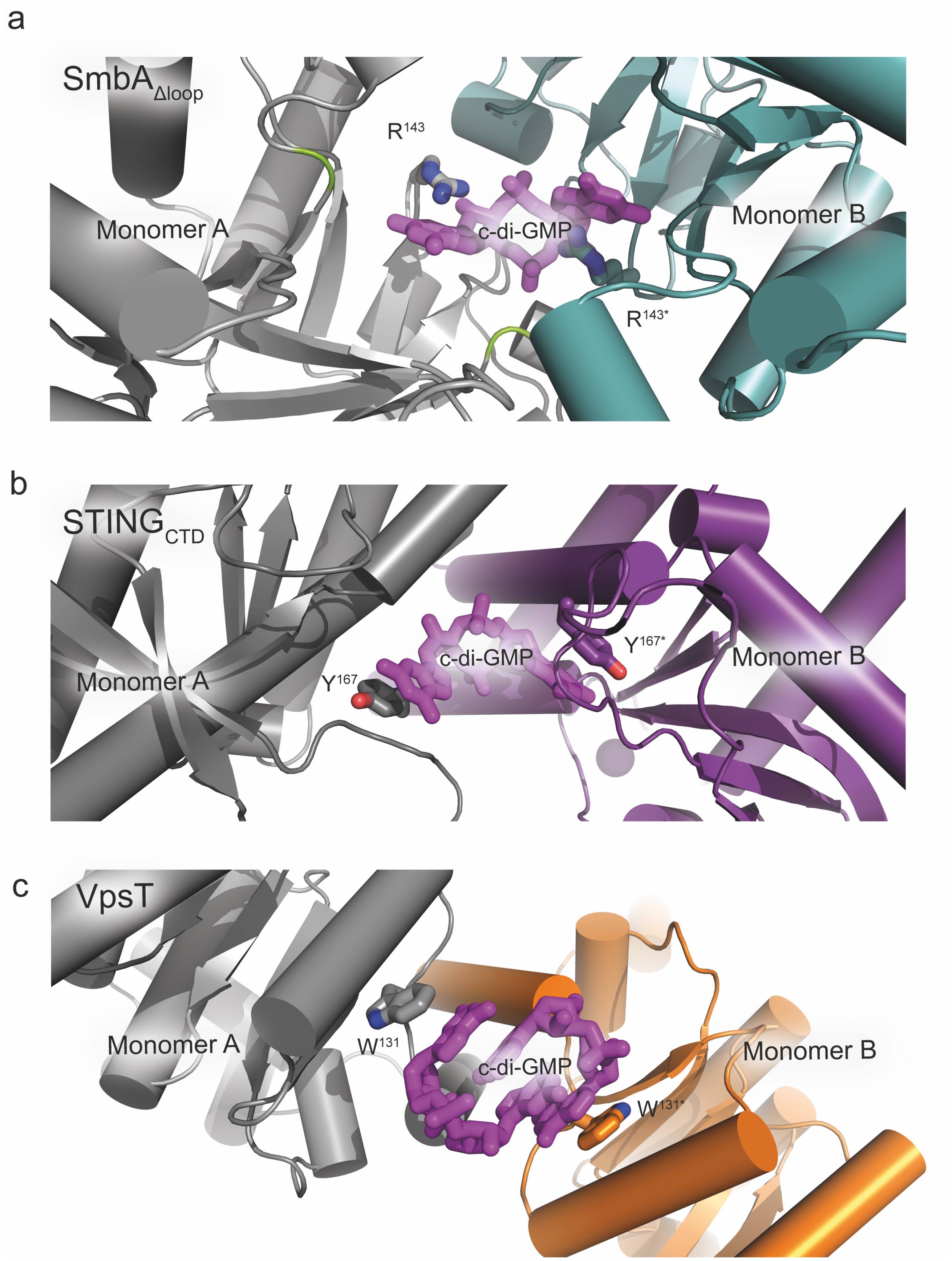

Figure S2. c-di-GMP bound at dimer interface found in STING and VpsT. c-di-GMP bind at the crystallographic dimer interface shown in the stick model (magenta). Residues involved in base stacking are shown in a stick. Monomers are colored differently. **(a)**$SmbA_{\Delta loop}$/c-di-GMP contact at the 2-fold crystallographic dimer interface. **(b)** STING c-di-GMP binding interface (PDB code-4F5Y). **(c)** VpsT dimerization interface (PDB code-3KLO).

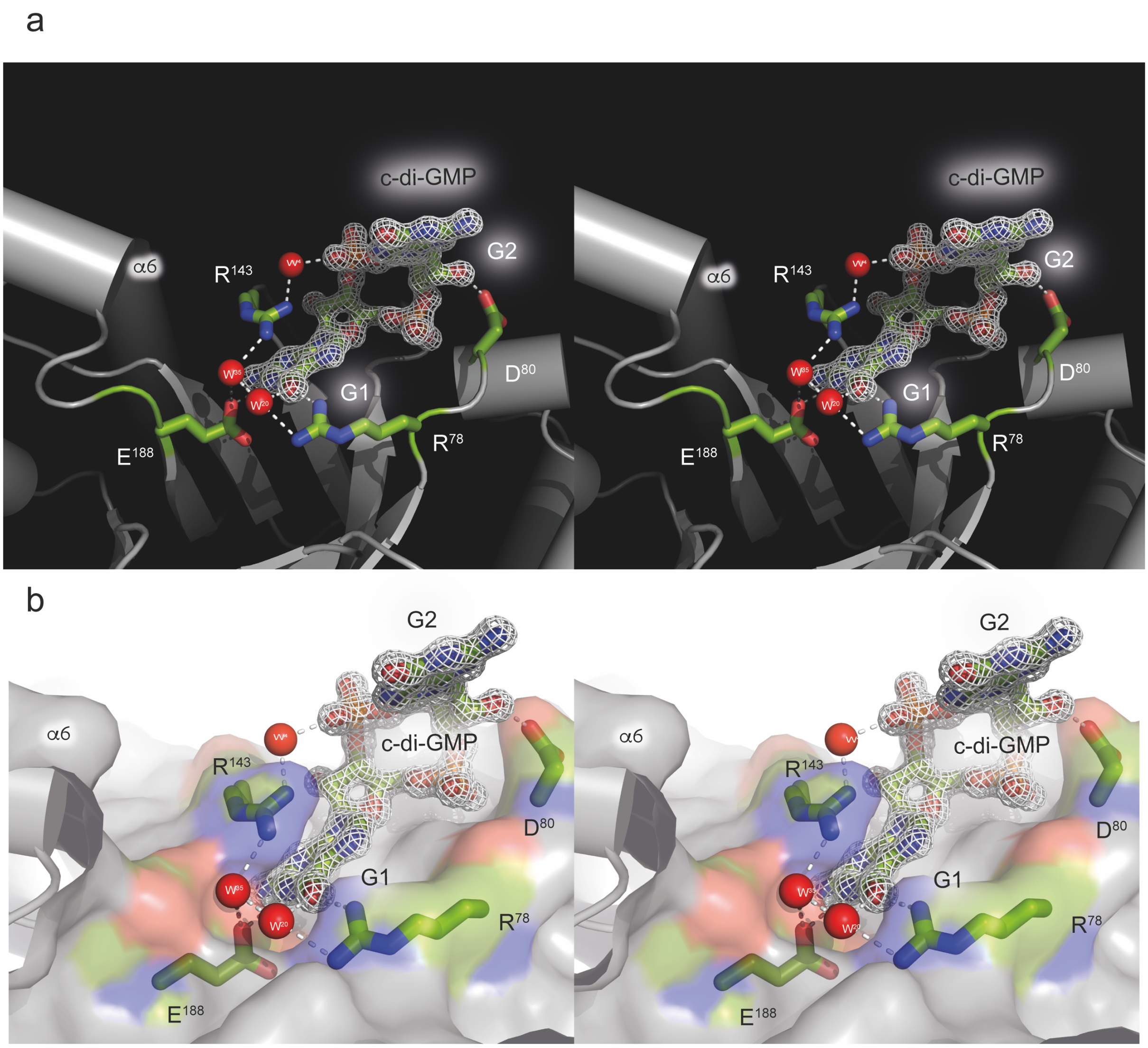

Figure S3. An expanded stereo view of the SmbA$_{\Delta loop}$ residues interacting with c-di-GMP.

(a) Stereo view of the 2Fo-Fc omit maps contoured at 1.2 σ. The molecular structure of c-di-GMP is embedded in the map. The colour code is similar to that in Fig. 3A. The R143 guanidinium group stacks very well with the guanyl base of c-di-GMP., while R78 is engaged in lateral H-bonding. **(b)** Stereo view of the c-di-GMP molecule drawn as surface representation (negatively charged atoms in red, positively charged atoms in blue and carbon atoms in green). Hydrogen bonds are marked by dotted lines in white.

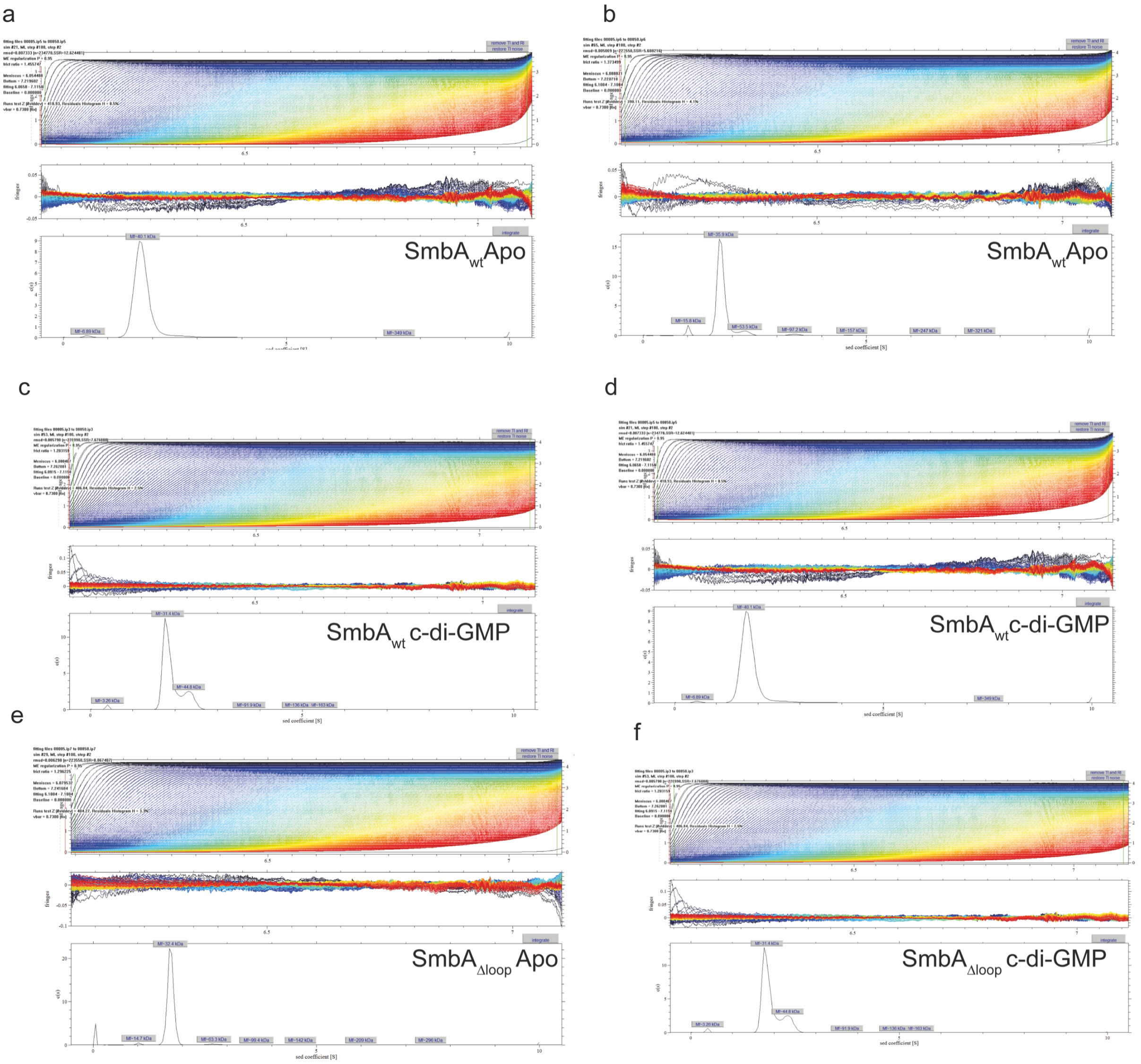

Figure S4. Absorbance data from the sedimentation velocity experiments of SmbA varients. Apo SmbA$_{wt}$ (**a** and **b**), SmbA$_{wt}$/c-di-GMP (**c** and **d**), Apo SmbA$_{\Delta loop}$ **(e)** and SmbA$_{\Delta loop}$/c-di-GMP **(f)** . At the top absorbance scans (symbols) and best-fit c(s) model at different points in time indicated by color temperature are shown. Bitmap and overlay of the residuals of the fit are show in the middle. At the bottom, c(s) sedimentation coefficient distribution showing peaks for monomer and some traces of higher oligomers.

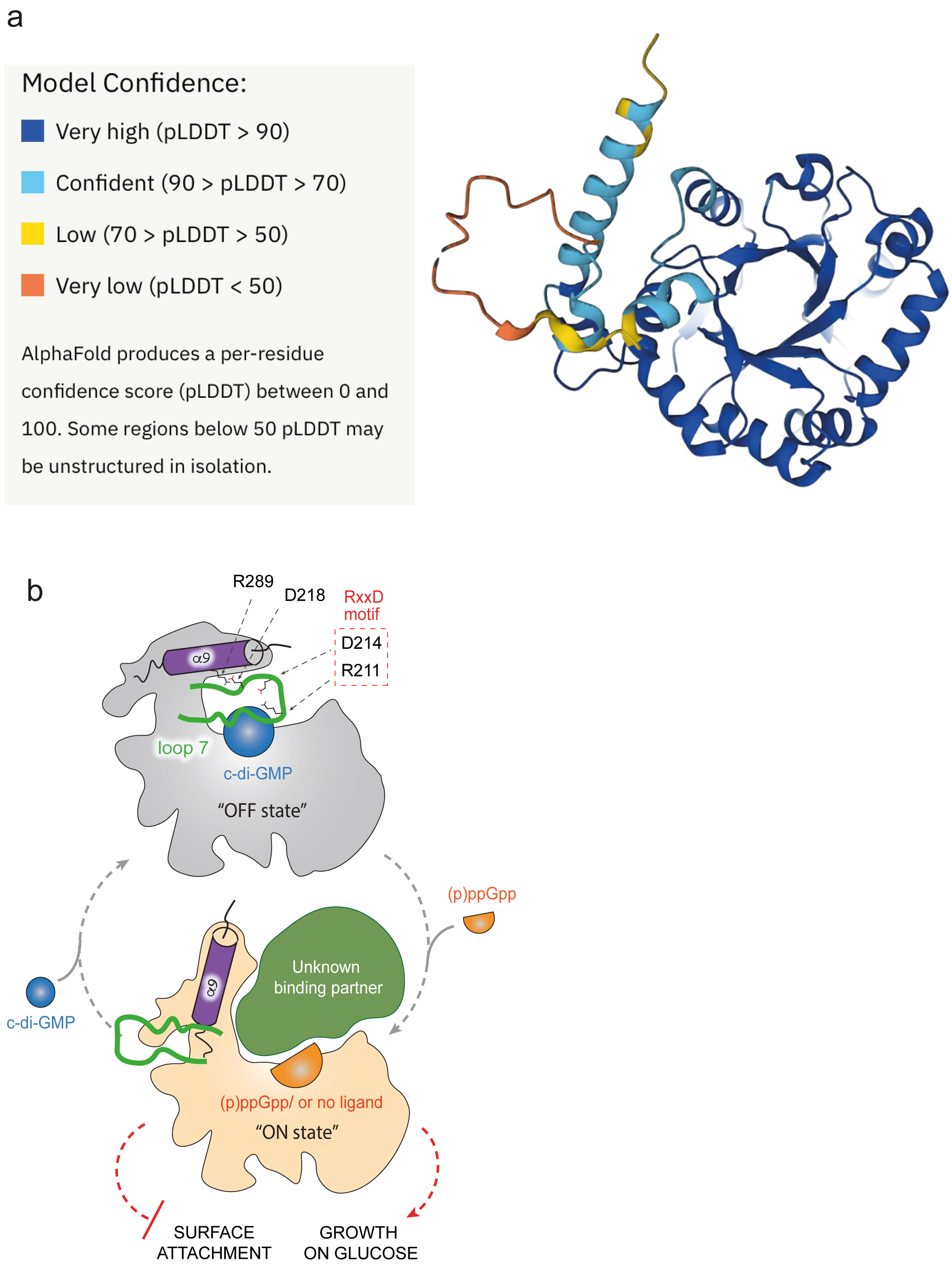

Figure S5. AlphaFold model of SmbA$_{wt}$ and proposed model of downstream signaling of SmbA. **(a)** AlphaFold model of SmbA from Uniprot # Q9A5E6. The confidence of the pLDDT is indicated by a color code. **(b)** Proposed model of downstream signaling by complex formation with an unidentified binding partner shown in dark green color.
